# Autologous Stem Cell Therapy for Chronic Lower Extremity Wounds: A Meta-Analysis of Randomized Controlled Trials

**DOI:** 10.3390/cells10123307

**Published:** 2021-11-25

**Authors:** Kuan-Ju Chiang, Li-Cheng Chiu, Yi-No Kang, Chiehfeng Chen

**Affiliations:** 1 School of Medicine, College of Medicine, Taipei Medical University, Taipei 110, Taiwan; b101104096@tmu.edu.tw (K.-J.C.); b101104072@tmu.edu.tw (L.-C.C.); 2 Department of Health Care Management, College of Health Technology, National Taipei University of Nursing Health Sciences, Taipei 112, Taiwan; 3 Evidence-Based Medicine Center, Wan Fang Hospital, Taipei Medical University, Taipei 116, Taiwan; 4 Research Center of Big Data and Meta-Analysis Center, Wan Fang Hospital, Taipei Medical University, Taipei 116, Taiwan; 5 Cochrane Taiwan, Taipei Medical University, Taipei 110, Taiwan; 6 Institute of Health Policy & Management, College of Public Health, National Taiwan University, Taipei 100, Taiwan; 7 Division of Plastic Surgery, Department of Surgery, Wan Fang Hospital, Taipei Medical University, Taipei 116, Taiwan; 8 Department of Public Health, School of Medicine, College of Medicine, Taipei Medical University, Taipei 110, Taiwan

**Keywords:** stem cell, chronic wounds, lower limb, lower extremity, diabetes mellitus foot, critical limb ischemia

## Abstract

Lower extremity chronic wounds (LECWs) commonly occur in patients with diabetes mellitus (DM) and peripheral arterial disease (PAD). Autologous stem cell therapy (ASCT) has emerged as a promising alternative treatment for those who suffered from LECWs. The purpose of this study was to assess the effects of ASCT on LECWs. Two authors searched three core databases, and independently identified evidence according to predefined criteria. They also individually assessed the quality of the included randomized controlled trials (RCTs), and extracted data on complete healing rate, amputation rate, and outcomes regarding peripheral circulation. The extracted data were pooled using a random-effects model due to clinical heterogeneity among the included RCTs. A subgroup analysis was further performed according to etiology, source of stem cells, follow-up time, and cell markers. A total of 28 RCTs (*n* = 1096) were eligible for this study. The pooled results showed that patients receiving ASCT had significantly higher complete healing rates (risk ratio (RR) = 1.67, 95% confidence interval (CI) 1.28–2.19) as compared with those without ASCT. In the *CD34*+ subgroup, ASCT significantly led to a higher complete healing rate (RR = 2.70, 95% CI 1.50–4.86), but there was no significant difference in the *CD34*− subgroup. ASCT through intramuscular injection can significantly improve wound healing in patients with LECWs caused by either DM or critical limb ischemia. Lastly, *CD34*+ is an important cell marker for potential wound healing. However, more extensive scale and well-designed studies are necessary to explore the details of ASCT and chronic wound healing.

## 1. Introduction

On the one hand, lower extremity chronic wounds (LECWs) commonly occur in patients with diabetes mellitus (DM) and peripheral arterial disease (PAD) [1,2,3]. The lifetime risk of a patient with diabetes developing a diabetic foot ulcer is 25%, and foot ulcers precede up to 85% of all lower-limb amputations in diabetes [4,5]. On the other hand, studies have shown that the prevalence of PAD in the general population is 3% to 10%, with 11.2% of those with PAD deteriorating to critical limb ischemia (CLI) each year [6]. CLI increases the risk of amputation as high as 15–20% at one year and also reduces patients’ life expectancies with mortalities typically exceeding 50% by five years [7].

LECWs are usually treated by necrotic tissue debridement, wet dressing, and nutritional support. Although diverse therapeutic approaches are available to manage chronic wounds, some have limited success and do not promote consistent, complete wound closure. Therefore, advanced treatment options for LECWs have become an immediate priority, and autologous stem cell therapy is one of the advanced treatments for LECWs. Actually, autologous stem cell therapies have emerged as a promising alternative treatment for those who suffer from LECWs [8,9]. Notably, stem cells can influence many pathophysiologic processes involved in the healing of ulcers, for instance, through stimulating tissue repair cells’ activities, increasing the synthesis of extracellular matrix, releasing growth factors, and promoting angiogenesis in the ischemic tissue [8,9]. The ischemic limbs in animal models had improved blood flow circulation after stem cell implantation in some reports [10,11,12]. In 2002, the first human trial suggested that bone marrow mononuclear cell implantation was safe and effective for therapeutic angiogenesis in patients with CLI. Consequently, it promoted complete ulcer healing and reduced the amputation rate [13]. Since then, there has been accumulating evidence indicating that autologous stem cell therapy (ASCT) was more effective than the standard treatment for LECWs [14].

Recent studies have proven that stem cell therapy could reduce the amputation rate; however, the relationship between chronic wounds that lead to amputation and stem cells has rarely been discussed in detail [15,16]. Although there have been some meta-analyses of stem cell therapy in treating chronic wounds, most of the studies have investigated one etiology at a time, such as DM or CLI alone [17,18]. One study indicated that updated randomized controlled trials (RCTs) were not included in those studies [19]. In addition, the effectiveness of stem cells derived from different sources also needs to be confirmed [20]. Therefore, our study aimed to explore the role of stem cells in the care of chronic wounds as the initial treatment to reduce subsequent amputations, and to provide high-quality evidence via a systematic review and meta-analysis of RCTs.

## 2. Materials and Methods

The present systematic review utilized the Preferred Reporting Items for Systematic Review and Meta-Analyses (PRISMA) guidelines and PROSPERO CRD42021248746 (https://www.crd.york.ac.uk/prospero/display_record.php?RecordID=248746 (accessed on 3 November 2021)). The primary objective of this study was to perform a comprehensive review of the therapeutic efficacy and safety of administration of autologous stem cells in patients with chronic lower extremity ulcers. The systematic review and meta-analysis of RCTs were conducted following the recommendations of the Cochrane Collaboration, and were reported according to the Preferred Reporting Items for Systematic Review and Meta-Analyses Protocols (PRISMA-P) guidelines.

### 2.1. Literature Search and Study Selection

The literature was searched using 3 databases, including PubMed, Embase, and Cochrane Controlled Trials Register to identify articles published from building the databases to December 2020. The search terms were (stem cell OR colony forming OR mother cell* OR “cell therapy”) AND (chronic wound OR ulcer* OR wound) AND chronic AND (lower limb OR leg OR foot OR ankle OR knee).

The studies were screened and retrieved independently by two authors (K-J.C. and L-C.C.) in the first round of the search to obtain a list of studies that appeared to be relevant to our review. If there were any disagreements on study selection, another author participated for the final decision.

Studies were included if they met all of the following criteria: (a) patients with LECWs, (b) received ASCT, (c) reported as randomized controlled trials (RCTs), (d) the control group received standard therapy with or without sham injections, and (e) identified as stem cells with further details. Studies were excluded if they were (a) non-human trials; (b) reviews, case reports, and other studies not designed as RCT; or (c) without retrievable full-length articles.

### 2.2. Data Extraction and Quality Evaluation

For each included study, data were extracted, including countries, numbers of participants, subject characteristics (age, sex), underlying causes of lower extremity wounds, type of ASCT, follow-up durations, injection methods, tumor markers, complete wound healing rates, total amputation rates, major amputation rates, as well as adverse events. The complete wound healing rate was commonly defined as full re-epithelialization of the wound surface without discernable exudate or drainage after treatment and within a study period. On the one hand, the total amputation rate was calculated based on the events of major and minor amputations. On the other hand, the major amputation rate was only calculated based on the events of major amputations. Two review authors (K-J.C. and L-C.C.) independently extracted the data from the literature and performed quality assessments according to the predefined inclusion criteria. Differences between the two authors were discussed with another author to reach a consensus.

Each included study was evaluated using the Cochrane Collaboration tool for assessing risk of bias [21]. This quality evaluating strategy addressed aspects including random sequence generation, allocation concealment, blinding of participants and personnel, blinding of outcome assessors, incomplete outcome data, and selective reporting. The studies were categorized into high, low, and unclear risk of bias for each aspect. The two authors (K-J.C. and L-C.C.) conducted quality evaluations independently, and had a consensus meeting with an experienced researcher if their evaluations were inconsistent.

### 2.3. Analysis and Statistics

The meta-analysis was performed with RevMan 5.4 software, as recommended by Cochrane Handbook for Systematic Review of Interventions [21]. A risk ratio (RR) with 95% confidence interval (CI) was used to measure the dichotomous data. The continuous data were pooled by weighted mean difference (MD) for measurement based on similar units; whereas, when measurements were different, a standardized mean difference (SMD) with 95% CI was used. The Forest plots were visually inspected for result inconsistencies. Heterogeneity among studies was measured using I-square statistics (values ≥50% indicating substantial statistical heterogeneity among the trials) and chi-square tests (with *p* < 0.05 representing heterogeneity). Due to clinical heterogeneity, a random effects model was used to estimate pooled effect. The subgroup analysis was carried out for measuring time points, etiology, routes of administration, and source of stem cells. Moreover, we further performed a subgroup analysis for *CD34*, since it is a cell marker expressed by an extensive range of cells such as hematopoietic stem cells, endothelial progenitor cells, epithelial progenitor cells, and mesenchymal stromal cells [22,23]. A sensitivity analysis was carried out for the primary outcome by removing potential outliers indicated by Cook’s distance based on a Gaussian mixture model. Publication bias was assessed using funnel plots and Egger’s regression test for primary outcome [24] The trim-and-fill method was further applied for exploring whether pooled results could be seriously affected by publication bias. These further analyses were carried out using R version 4.1.0 via RStudio version 1.4.

## 3. Results

**Figure 1:**
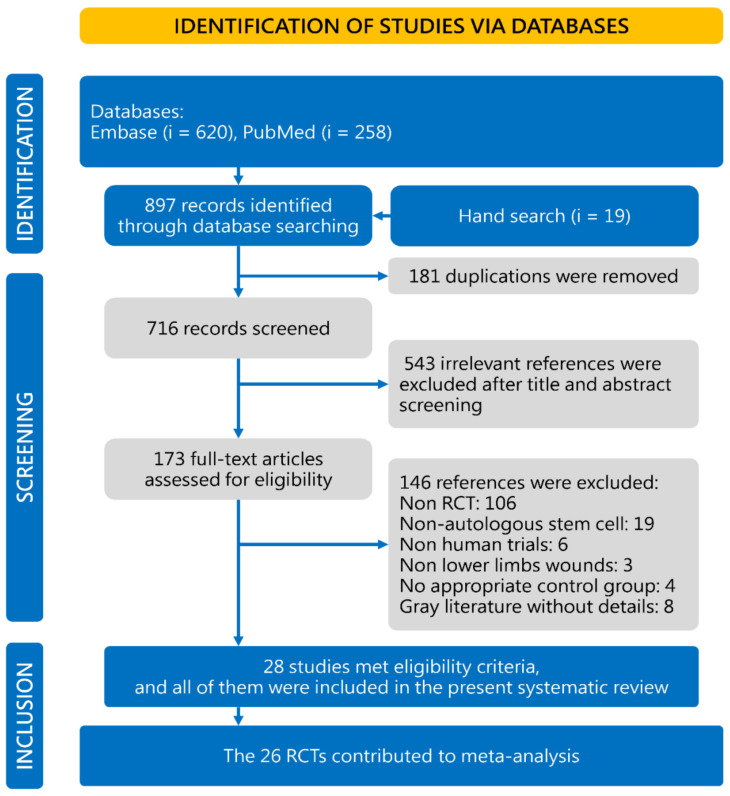
Study selection flowchart according to the PRISMA guidelines. RCT, randomized controlled trial.

Concisely, a total of 716 studies were identified in the initial database search; 543 articles were excluded because they were not relevant to our study’s objective, according to our screening process outlined in Figure 1. Among the 173 potentially relevant studies, 28 RCTs matched the inclusion criteria for the current meta-analysis [15,25,26,27,28,29,30,31,32,33,34,35,36,37,38,39,40,41,42,43,44,45,46,47,48,49,50,51]. There were 146 studies further excluded: 106 studies were not RCTs, 19 studies did not use ASCT, 6 studies were non-human trials, 3 studies did not include lower extremity wounds, 4 studies used control groups that did not receive standard treatment with or without sham injections, and 8 studies were not retrievable full-length articles. In addition to the references in the flowchart, another 63 registry records in Cochrane Controlled Trials Register (based on primary search strategy in title abstract keyword) were also screened, while there was no additional RCTs on this topic.

### 3.1. Characteristics of Included Studies

The general characteristics of the included studies are listed in Table 1. The 28 RCTs (*n* = 1096) were conducted in Africa, America, Asia, and Europe, and were published between 2005 and 2020. Recruitment numbers in each RCT ranged from 10 to ~160 patients [45,49], with the mean age from 60 to 74, except for three studies from India with younger ages from 40 to 58 [26,29,32]. The follow-up periods ranged from one month to three years after cell implantation. We categorized the follow-up times into three groups: (1) short term, with less or equal to three months in fifteen studies; (2) medium term, with more than three months to less than twelve months in nine studies; and (3) long term with more or equal to twelve months in six studies, respectively. In addition to the cell therapy groups, the control groups with conventional treatments included a placebo, such as saline or autologous peripheral blood injection, and a standard wound care regime. Regarding the etiology of LECWs, patients with CLI were included in eighteen RCTs, peripheral arterial occlusive disease (PAOD) in three RCTs, DM in eleven RCTs, and venous leg ulcers (VLU) in two RCTs.

The RCTs used three sources of autologous stem cells: (a) bone marrow-derived stem cells (BMSCs) in seventeen RCTs, (b) peripheral blood-derived stem cells (PBSCs) in five RCTs, and (c) adipose-derived stem cells (ASCs) in three RCTs. Cells were implanted into the affected limb via intramuscular (IM) route in twenty-one studies, intraarterial (IA) route in four studies, and topical application in two studies.

The risk of bias for the included studies was evaluated by the Cochrane assessment tool and are summarized in Table 2. Twelve of the included RCTs were double-blinded, placebo-controlled studies. Six of the studies were at high risk of bias for blinding of participants and personnel. One study was at a high risk of bias for incomplete outcomes according to the Cochrane Collaboration tool. Fourteen studies reported methods of random sequence, and seven studies reported the details of allocation concealment. Details of dropouts and withdrawals were reported in twenty-five studies.

### 3.2. Complete Wound Healing

**Figure 2:**
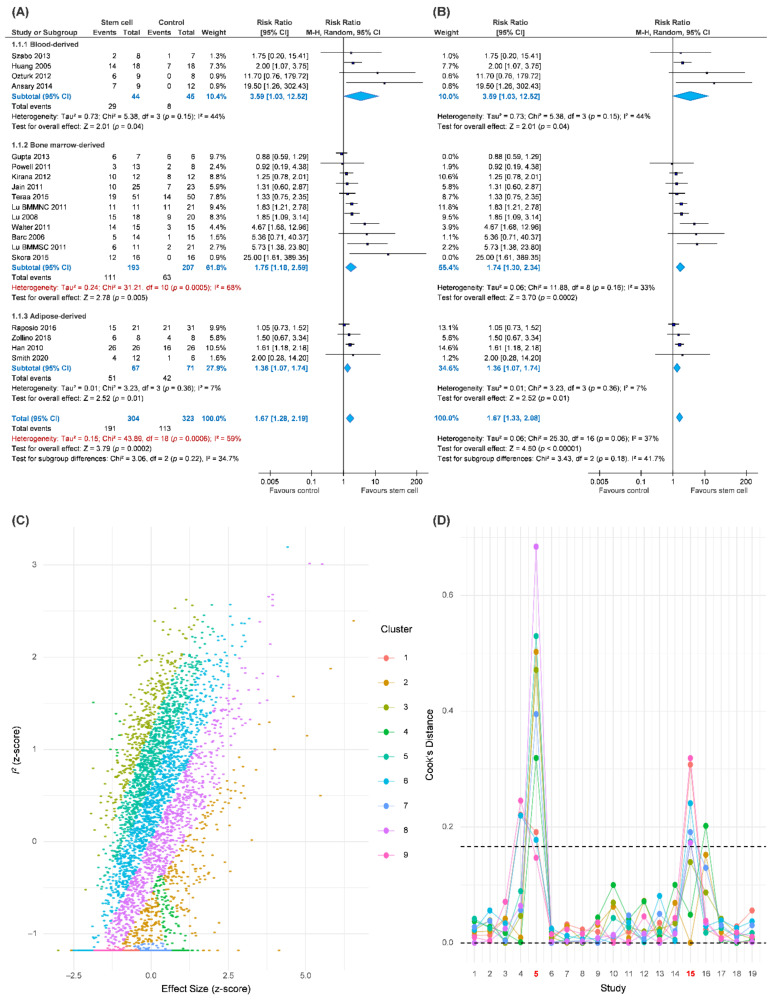
Pooled analysis of complete wound healing with: (**A**) Forest plot using all data; (**B**) forest plot of sensitivity analysis; (**C**) exploring heterogeneity by Gaussian mixture model; (**D**) Cook’s distance based on a Gaussian mixture model for identification of potential outliers.

**Figure 3:**
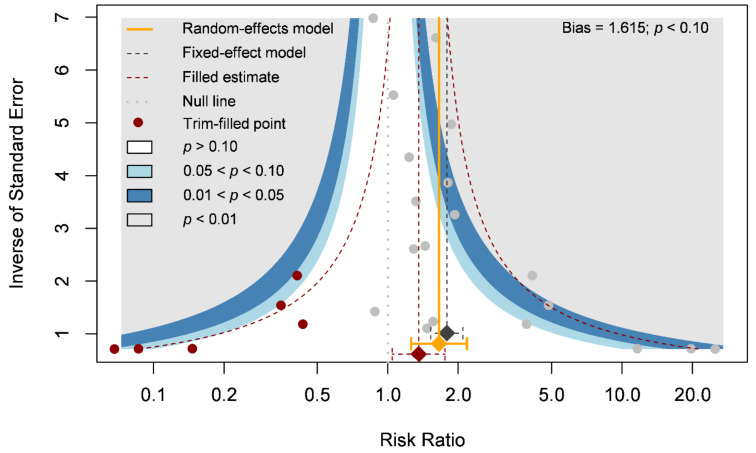
Funnel plot of complete wound healing.

Our primary outcome was complete wound healing in nineteen comparisons. With 304 patients treated with the ASCT and 323 patients assigned to the control groups, we investigated the effects of ASCT on the healing of LECWs (Figure 2A). Complete wound healing was defined as 100% re-epithelialization as judged by the treating podiatrist and clinician, with no need for further dressing therapy [47]. Overall, the results indicated that ASCT was associated with a significant increase in the complete wound healing rate as compared with that observed in the control groups. (RR = 1.67, 95% CI 1.28–2.19, *p* < 0.001). However, high heterogeneity existed in the pooled result (I-square = 59%), and the heterogeneity was reduced in the sensitivity analysis (I-square = 37%, Figure 2B). According to a Gaussian mixture model (Figure 2C), the potential outliers were identified according to Cook’s distance (Figure 2D). The funnel plot was asymmetric, although the result seemed to be not seriously affected due to similar trends and a significant effect of ASCT after the analysis with the trim-and-fill method (Figure 3). We tried to determine whether autologous stem cells from different sources were associated with better complete healing rates of LECWs. Therefore, our subgroup analyses consisted of different stem cell sources, etiology of chronic wounds, mean follow-up time, and cell markers, from the available information from 19 RCTs.

The results of the subgroup analyses of different stem cell sources all showed significantly better complete wound healing than the control groups, for example, PBSCs (RR = 3.59, 95% CI 1.03–12.52, *p* = 0.04), BMSCs (RR = 1.75, 95% CI 1.18–2.59, *p* = 0.005), and ASCTs (RR = 1.36, 95% CI 1.07–1.74, *p* = 0.01).

The results of the subgroup analyses of chronic wound etiology revealed that ASCT significantly and similarly increased the complete wound healing rates in patients with either DM (RR = 1.57, 95% CI 1.19–2.08, *p* = 0.002) or CLI (RR = 2.53, 95% CI 1.01–6.31, *p* < 0.05) as compared with the control groups. In contrast, in the VLU subgroup, the ASCT did not reach significant differences as compared with the control groups. (VLU, RR = 1.50, 95% CI 0.67–3.34).

The mean follow-up time of complete wound healing was reported in 17 studies. Chronic wounds are recognized as unhealed wounds that last over 3 months after appropriate treatment. Therefore, we categorized the observation durations into short-term follow-up (<3 months), medium-term follow-up (6 months), and long-term follow-up (≥1 year). The result of the complete wound healing rate in the short-term follow-up subgroup (RR = 1.94, 95% CI 1.44–2.62, *p* < 0.001) was similar to the medium-term follow-up subgroup (RR = 1.87, 95% CI 1.05–3.35, *p* = 0.03). However, no significant difference was observed in the long-term follow-up subgroup (RR = 1.04, 95% CI 0.83–1.31).

Cell markers were reported in 11 studies in total. ASCT expressing *CD34* positive marker was included in eight studies. Conversely, *CD34* negative marker was reported in three studies. The result showed that the *CD34* positive marker was more beneficial than the *CD34* negative marker concerning the rate of complete wound healing. While the former had a significantly higher complete wound healing rate as compared with the control groups (*CD34*+, RR = 2.70, 95% CI 1.50–4.86, *p* < 0.001), the latter had no significant difference (*CD34*−, RR = 1.78, 95% CI 0.65–4.89).

The result demonstrated that intramuscular autologous stem cell transplantation was beneficial to the complete wound healing rate (RR = 1.84, 95% CI 1.20–2.82, *p* = 0.005). In addition, there were also two studies that used topical application of stem cells on wound beds, which also indicated significant improvement (RR = 1.61, 95% CI 1.19–2.18, *p* = 0.002). In contrast, intra-arterial transplantation showed no significant difference as compared with the control groups (RR = 1.28, 95% CI 0.75–2.17).

### 3.3. Amputation and Peripheral Circulation

Other outcomes including total amputation rate, major amputation rate, ankle brachial index (ABI), and TcPO2 were extracted from all of the 16 studies. Major amputation was defined as an amputation through or above the ankle joint [41]. Meanwhile, total amputation included both below and above-the-knee amputation [28]. For total amputation rate and major amputation rate, the result showed that ASCT had a trend toward lower total (RR = 0.54, 95% CI 0.39–0.74, *p* < 0.001) and major (RR = 0.66, 95% CI 0.44–0.98, *p* = 0.04) amputation as compared with the control groups with a statistically significant difference (Table 3). The results also indicated that cell therapy significantly improved the ABI (MD = 0.12, 95% CI 0.06–0.18, *p* < 0.001) and TcPO2 (MD = 3.65, 95% CI −0.04–7.34, *p* = 0.05).

### 3.4. Safety

A total of 5 out of 28 studies reported adverse events related to ASCT. Complications considered to be related to the treatment included: five patients receiving G-CSF complained of mild bone pain [25]; one patient experienced moderate cellulitis after bone marrow aspiration and another patient developed a severe localized infection of the treated wound [42]; one patient experienced moderate hypotension during cell mobilization and another patient experienced severe worsening of CLI in the target leg after injection [36]; one patient presented peripheral edema at the injection site [46]; and one patient had inguinal hematoma as a result of intra-arterial infusion [49].

## 4. Discussion

### 4.1. Key Findings

The results indicated that ASCT significantly improved the complete wound healing rate as compared with standard treatment for LECWs. Regarding the subgroup analysis of the primary outcome, the results showed that PBSCs, BMSCs, ASCs, wounds caused by either DM or CLI, short- or medium-term follow-up, IM application, and *CD34*+ cell marker had a better outcome of complete wound healing rate using ASCT. Studies showed consensus results that ASCT could promote the healing of LECWs [19].

The evaluation of the robustness of our findings was affected by several studies. One RCT presented poor wound healing among all 17 quantitative RCTs [29], making the outcomes of complete wound healing in CLI and BMMSC subgroups less significant. Moreover, two studies affected the ASC and IA subgroup results [44,49], respectively, by their larger sample size. However, most of the outcomes remained stable after the evaluation of the sensitivity test.

In addition, our results showed significantly increased values of ABI and TcPO2 levels, which were meaningful to confirm the improvements of total and major amputation rates and wound healing rate. ABI and TcPO2 were both noninvasive methods to measure tissue perfusion and effectively reflect the metabolic state of lower limbs [52,53,54]. Moreover, they were currently used in clinical practice in the management of the diabetic foot or PAOD; in particular, they were important in determining amputation level, wound healing evaluation, and revascularization procedures [53,55,56,57]. Several clinical trials conducting stem cell therapy were consistent with our results [58,59,60]. Note that both total and major amputation rates had significantly decreased in the stem cell therapy groups as compared with the control groups. ASCT provided a better prognosis for patients with LECWs. In addition, the data of ABI and TcPO2 suggested that this procedure could have improved long-term effects [61].

### 4.2. Potential Mechanism

In a recent study, Wu (2007) discussed the mechanism and effects of BMMSCs among mice models that resulted in capillary density enhancement and significantly increased amounts of angiopoietin-1 and VEGF-α in wounds, but not angiopoietin-2. VEGF plays a key role in angiogenesis by stimulating endothelial cell proliferation, migration, and organization into tubules [62,63,64]. Moreover, VEGF increases circulating endothelial progenitor cells [62]. In addition to the angiogenic factors, macroscopic visualization of blood vessels in wounds at 7 days showed blood vessels surrounding the wounds, but were limited in the wounds. In contrast, in wounds of the BMMSC group, vessels and their fine branches extended into the wounds and formed networks. Briefly, BMMSCs engrafted in the wound release proangiogenic factors, which may be partially responsible for MSC-mediated enhanced angiogenesis [65]. The significant increase in the values of ABI and TcPO2 in our study corresponded to those in Wu’s study, which represented objective evidence of improvement in angiogenesis caused by stem cells. Furthermore, we presumed that after using stem cell treatment, the effect of angiogenesis would occur within three months. This hypothesis was proven by the observation of a higher density of capillaries at 14 days in Wu’s study and the significant increase in ABI and TcPO2 during the short-term follow-up in our study. Angiogenesis, which plays an important role in chronic wound healing [66], even affects the incidence of amputation rate, and could become the most important advantage of stem cell therapy [67].

### 4.3. Source of Heterogeneity

#### 4.3.1. Source of Stem Cells

Our systemic review proved that different sources of stem cells such as PBSCs, BMSCs, and ASCs had effective functions on complete wound healing, in agreement with a current meta-analysis [68]. Recent studies have reported that the implantation of PBSCs into ischemic limbs benefits through two mechanisms [69]. First, PBSCs promote angiogenesis and endothelial progenitor cell migration to the ischemic area. Second, PBSCs secrete angiogenic factors that promote neovascularization and replenish damaged vascular cells [69]. When it comes to BMSCs, which can be divided into bone marrow-derived mesenchymal stem cells (BMMSCs) and bone marrow-derived mononuclear cells (BMMNCs), in general, they have both been proven to accelerate angiogenesis and wound healing in many preclinical studies [11,70,71,72,73,74]. In the re-epithelialization phase of wound healing, ASCs play the role of angiogenesis, growth factor secretion. They also allow human dermal fibroblast proliferation through direct cell contact and paracrine activation [75]. Therefore, PBSCs, BMSCs, and ASCs were all proven to be beneficial to wound healing rate. However, after considering the risk of bleeding and anesthesia associated with bone marrow aspiration [76], we suggest that PBSCs and ASCs should be the preferred type of stem cell due to the less invasive harvesting process and abundant blood and fat as compared with BMSCs.

#### 4.3.2. Etiology of Wounds

Our subgroup analysis indicated that ASCT was statistically effective in both DM-caused and CLI-caused LECWs. Preclinical and clinical studies have proven that ASCT can accelerate the healing of DM wounds [77,78], and can improve microvascular regeneration around wound areas [61], while other studies have indicated the effectiveness of stem cells on CLI wounds, which was consistent with our results [8,79]. However, stem cell therapy for VLUs-caused wounds still remains unclear due to the small sample size (1 RCT, *n* = 16). Collectively, ASCT can maximize wound healing effectiveness in DM and CLI patients. Additionally, more large-scale and well-designed RCTs focused on stem cell therapy against VLUs would be required to confirm and update our results.

#### 4.3.3. Mean Follow-Up Time

The subgroup analysis indicated significant healing rates in both short-term follow-up and medium-term follow-up. Our result was consistent with recent studies [78,80]. In other words, wound healing progressed rapidly in the first six months after stem cell application. Our findings were also in agreement with a previous study. After topical application of BMSCs in chronic wounds (ulcer duration >1 year), all treated wounds began to close within 2–4 weeks, and after 16–20 weeks, wounds closed completely [81]. However, one study showed the opposite result which might be due to exclusively different intervention and follow-up times [82]. Many factors can affect the wound healing process. We presumed that once the wounds have not healed entirely within six months after ASCT, it means that ASCT has achieved its optimal effect. Alternative therapies should be taken into consideration.

#### 4.3.4. Cell Markers

We conducted a subgroup analysis of *CD34* cell markers and identified *CD34* positive groups with a better complete healing rate. Although the function of *CD34* as a surface antigen is still under debate, studies have proven that *CD34* was involved in interactions with cell surface adhesion molecules, cell proliferation, and regulation of differentiation [83,84]. On the one hand, preclinical studies have demonstrated the potency of *CD34* positive (*CD34*+) cells for therapeutic neovascularization and improved tissue perfusion and function by local delivery in myocardial and limb ischemia models [85,86]. On the other hand, *CD34* negative stem cells have been proven to reconstitute hematopoietic cells [87,88,89,90,91,92,93], and were even assumed to have the potency to generate whole organ systems without limitation [94]. According to the results of previous studies and our meta-analysis, we propose that *CD34*+ stem cells significantly influence wound healing in LECWs, while *CD34*− stem cells have less effect.

#### 4.3.5. Route of Administration

In our included RCTs, intramuscular injection (IM) turned out to be the most common route of ASCT administration. Aside from intramuscular injection, intra-arterial injection and topical application were also conducted in ASCT. To find out the most useful and profitable route, we ran another subgroup analysis. Our result showed that the intramuscular subgroup had a significantly higher complete wound healing rate than the intra-arterial subgroup. The explanations of our result are as followS: First, in patients with microvascular complications of DM or arterial occlusion of CLI, the peripheral perfusion is compromised by their disease’s nature. Intramuscular administration avoids this problem by transporting the cells closer to wound sites [95]. Secondly, muscle tissue supports intramuscular-injected stem cells with nutrients and oxygen, which benefits stem cells’ survival and improves functions inside the human body [96]. Lastly, studies have shown that intravascular administration could trap stem cells in the lungs, leading to pulmonary embolism. Therefore, intramuscular injection seems to be a rather safe choice for administration [97,98]. Even though no adverse events of pulmonary embolism or other pulmonary symptoms have been reported in our included RCTs so far, the side effect is still a safety concern. The two included studies with topical application were both using ASCs and fibrinogen plus PRP as a cell carrier to enable the stem cells to graft on wounds longer [99,100,101]. Topical application provides cell metabolism, migration, and differentiation. Moreover, it can stimulate extracellular matrix secretion and tissue regeneration [102,103]; therefore, shortening treatment time and improving the survival rate of transplants, with better clinical application value [104,105,106]. Although most topical applications were used in allogeneic stem cell therapy, other studies’ results were consistent with our systemic review and meta-analysis [78,107]. Therefore, we suggest intramuscular or topical pathways to administer stem cells for safety and efficacy consideration. However, more studies comparing different applications of autologous stem cells with large sample sizes would be required to confirm our result.

### 4.4. Limitations

There were some limitations in our study. First, most trials had a high or unclear risk of bias, so that the trials may be underpowered. Some RCTs claimed “random” in the content but did not report the specific randomization method. Some RCTs did not use allocation concealment or blinding methods. Second, several studies had a smaller sample size and limited details of outcomes, such as adverse events. Third, we only included studies in English, which may lead to publication bias. Lastly, we mainly focused on the analysis of subjective clinical outcomes this time; assessment of patients’ perspectives could be taken into consideration in the future.

## 5. Conclusions

Autologous stem cell therapy can significantly promote wound healing in patients with chronic lower extremity wounds. PBSCs, ASCs, or BMSCs, especially BMMNCs were proven to be beneficial to wound healing rate. However, PBSCs and ASCs should be the preferred types of stem cells. In addition, stem cell therapy can produce the optimal effect in either DM or CLI patients through intramuscular injection. Lastly, *CD34*+ is an important tumor marker for potential wound healing. Selecting optimal therapy for non-healing wounds is contingent on patients’ variables, such as wound etiologies, as well as processing variables, including source of stem cell, route of administration, and cell markers. However, more extensive scale and well-designed clinical studies are necessary to substantiate a wider scope of application for stem cell therapy in treating chronic wounds.

## Figures and Tables

**Table 1:** Characteristics of trials included in the synthesis.

Author/Year Published	Male % (I/C)	Age (I/C)	Type of Cell Intervention	Route (I/C)	Follow-Up (Months)	*CD34* (+/−)	Relevant Outcome
Huang, 2005	9(64%)/9(64%)	71.1/70.9	PBMNC/NCT	IM	3	+	①②④
Arai, 2006	11(84%)/7(58%)	62/68	BMMNC/NCT	IM	1	+	④⑤
Barc, 2006	Unclear	Unclear	BMMNC/NCT	IM	3 and 6	+	①②
Lu, 2008	11(50%)/15(65%)	66.6/65.5	BMMSC/NCT	IM	3	–	①②④
Dash, 2009	Unclear	40	BMMSC/NCT	IM+Topical	3	–	No data
Prochazka, 2010	36(86%)/42(78%)	66.2/64.1	BMSC/NCT	IA	4	+	②
Han, 2010	15(58%)/14(54%)	66.5/68.4	ASC/NCT	Topical	2		①
Lu, 2011	7(39%)/8(42%)	63/65	BMMSC/BMMNC/NCT	IM	6	–	①②④⑤
Jain, 2011	17(68%)/15(63%)	54/58	BMSC/NCT	IM+Topical	3		①
Walter, 2011	16(84%)/13(62%)	64.4/64.5	BMMNC/NCT	IA	3 and 6	+	①③④⑤
Powell, 2011	25(78%)/8(57%)	68.8/65.9	BMSC/NCT	IM	6 and 12		①③
Iafrati, 2011	23(68%)/9(64%)	72.5/65.7	BMSC/NCT	IM	3		②③
Losordo, 2012	5(71%)/8(89%)/6(50%)	61.8(low)/69.7(high)/67.1	PB-*CD34*+/NCT	IM	6 and 12	+	②③
Ozturk, 2012	16(80%)/13(65%)	71.9/70.8	PB-*CD34*+/NCT	IM	3	+	①②④⑤
Kirana, 2012	9(75%)/10(83%)	68.5/70.9	BM-MNC/TRC	IM or IA	13		①③
Szabo, 2013	8(80%)/5(50%)	60.6/63.0	PBSC/NCT	IM	1 and 3	+	①②③⑤
Li, 2013	23(76%)/22(79%)	61/63	BMMNC/NCT	IM	6		③
Gupta, 2013	Unclear	43/47.6	BMMSC/NCT	IM	6 and 24	–	①②③④
Mohammadzadeh, 2013	Unclear	63.5/64.2	PBSC/NCT	IM	3		②③④
Raval, 2014	6(86%)/2(66%)	65/85	PB-CD133+/NCT	IM	12		②③
Ansary, 2014	9(72%)/8(66%)	50.5/61.7	PBMNC/NCT	IM	3	+	①②④
Teraa, 2015	57(70%)/51(65%)	69/65	BMMNC/NCT	IA	2 and 6	+	①②③④
Skora, 2015	11(69%)/10(38%)	66.7/68.3	BMMNC/NCT	IM	3	+	①②④
Raposio, 2016	11(69%)/10(42%)	70.7/74.5	ASC+ PRP/NCT	IM	18		①
Pignon, 2017	13(72%)/18(90%)	72/65	BMMNC/NCT	IM	6 and 12	+	②③
Zollino, 2018	5(63%)/5(63%)	74/68	ASC/NCT	IM	6	+	①
Lu, 2019	Unclear	≧64	BMMSC/BMMNC/NCT	IM	36		No data
Smith, 2020	5(92%)/6(100%)/4(66%)	60.2/55.2	ASC+PRP/ASC/NCT	Topical	3		①

ASC, adipose stem cell; BMMNC, bone marrow-derived mononuclear cell; BMMSC, bone marrow-derived mesenchymal stem cell; BMSC, bone marrow-derived stem cell; IA, intraarterial; I/C, intervention group/control group; NCT, non-cell therapy; PLA, human processed lipoaspirate cells; PBSC, peripheral blood-derived stem cell; TRC, tissue repair cells (expansion of bone marrow cells, *CD90*+); VLUs; venous leg ulcers; ①, complete healing rate; ②, total amputation rate; ③, major amputation rate; ④, ankle brachial index; ⑤, TcPO2.

**Table 2:** Risk of bias evaluation.

Study	Bias 1	Bias 2	Bias 3	Bias 4	Bias 5	Bias 6
Huang, 2005	Low	Unclear	High	Unclear	Low	Low
Barc, 2006	Low	Unclear	High	Unclear	Low	Low
Arai, 2006	Low	Unclear	High	Unclear	Low	Low
Lu, 2008	Unclear	Unclear	Unclear	Unclear	Low	Low
Dash, 2009	Low	Low	High	Unclear	Low	High
Han, 2010	Low	Low	Low	Unclear	Low	Unclear
Prochazka, 2010	Low	Unclear	High	Unclear	Low	Low
Jain, 2011	Low	Low	Low	Unclear	Low	Unclear
Lu, 2011	Low	Unclear	Low	Low	Low	High
Walter, 2011	Low	Unclear	Low	Low	Low	High
Powell, 2011	Low	Unclear	Low	Low	High	Low
Iafrati, 2011	Unclear	Low	Low	Low	Low	Low
Kirana, 2012	Low	Unclear	Unclear	Unclear	Low	Unclear
Losordo, 2012	Unclear	Unclear	Low	Low	High	High
Ozturk, 2012	Low	Low	High	High	Low	Low
Gupta, 2013	Low	Low	Low	Low	Low	Low
Mohammadzadeh, 2013	Low	Unclear	Unclear	Unclear	Low	Low
Li, 2013	Low	Unclear	High	Unclear	Low	Low
Szabo, 2013	Low	Unclear	Unclear	Unclear	Low	Low
Raval, 2014	Unclear	High	Low	Low	Low	Low
Ansary, 2014	Unclear	Unclear	Low	Low	Low	Low
Skora, 2015	Low	Unclear	High	Low	Low	Low
Teraa, 2015	Low	Low	Low	Low	High	Low
Raposio, 2016	Unclear	Unclear	Unclear	Unclear	Low	Unclear
Pignon, 2017	Low	Unclear	Low	Low	Unclear	Unclear
Zollino, 2018	Low	Unclear	Unclear	Unclear	Low	Unclear
Lu, 2019	Unclear	Unclear	Low	Unclear	Low	Unclear
Smith, 2020	Low	Low	High	Low	Low	Unclear

Bias 1, random sequence generation (selection bias); Bias 2, allocation concealment (selection bias); Bias 3, blinding of participants and personnel (performance bias); Bias 4, blinding of outcome assessment (detection bias); Bias 5, incomplete outcome data (attrition bias); Bias 6, selective outcome reporting (reporting bias).

**Table 3:** Summary of findings of amputation and peripheral circulation.

	Outcome	Total Amputation Rate			Major Amputation Rate			Ankle Brachial Index			TcPO2		
Subgroup		Studies/Cases	RR (95% CI)	I^2^	Studies/Cases	RR (95% CI)	I^2^	Studies/Cases	RR (95% CI)	I^2^	Studies/Cases	RR (95% CI)	I^2^
Etiology													
CLI		11/499	0.61 (0.45, 0.83)	22%	10/462	0.66 (0.44, 0.99)	0%	6/201	0.18 (0.04, 0.32)	83%	3/61	3.46 (0.23, 6.68)	0%
DM + CLI		5/227	0.28 (0.11, 0.71)	1%	2/45	0.76 (0.02, 28.96)	70%	5/235	0.09 (0.01, 0.160	75%	2/122	4.05 (−3.73, 11.84)	43%
Intervention													
Blood-derived		7/186	0.41 (0.25, 0.69)	1%	4/75	0.40 (0.12, 1.28)	31%	4/132	0.28 (0.08, 0.49)	82%	2/60	6.92 (−3.87, 17.71)	83%
Bone marrow-derived		9/540	0.62 (0.42, 0.890	28%	8/432	0.73 (0.46, 1.16)	0%	7/304	0.06 (0.01, 0.10)	57%	3/123	2.50 (−0.87, 5.88)	2%
Follow-up time													
Short term (≦3 m)		10/380	0.42 (0.28, 0.630	0%	4/129	0.47 (0.11, 2.06)	51%	9/342	0.13 (0.06, 0.20)	81%	5/183	3.65 (−0.04, 7.34)	48%
Medium term (6 m)		4/320	0.62 (0.38, 1.02)	50%	5/328	0.62 (0.39, 1.01)	0%	1/77	0.03 (−0.09, 0.15)	-	-	-	-
Long term (≧12 m)		2/26	1.70 (0.52, 5.500	0%	3/50	1.349 (0.38, 4.69)	0%	1/17	0.17 (0.03, 0.31)	-	-	-	-
Tumor marker													
*CD34*+		9/476	0.54 (0.37, 0.79)	31%	4/248	0.76 (0.3, 1.93)	39%	7/271	0.16 (0.05, 0.28)	79%	4/101	5.78 (0.72, 10.82)	53%
*CD34*–		3/139	0.34 (0.04, 2.91)	59%	1/20	1.00 (0.17, 5.77)	-	3/144	0.06 (−0.02, 0.14)	79%	1/82	0.10 (−4.29, 4.50)	0%
Overall		16/726	0.55 (0.40, 0.75)	25%	12/507	0.66 (0.44, 0.98)	0%	11/436	0.12 (0.06, 0.18)	78%	5/183	3.65 (−0.04, 7.34)	87%

CI, confidence interval; CLI, critical limb ischemia; DM, diabetes mellitus; RR, risk ratio.

## Data Availability

PROSPERO CRD42021248746 (https://www.crd.york.ac.uk/prospero/display_record.php?RecordID=248746 (accessed on 2 November 2021)).
